# Diverse Roles of Ethylene in Regulating Agronomic Traits in Rice

**DOI:** 10.3389/fpls.2017.01676

**Published:** 2017-09-26

**Authors:** Cui-Cui Yin, He Zhao, Biao Ma, Shou-Yi Chen, Jin-Song Zhang

**Affiliations:** ^1^State Key Lab of Plant Genomics, Institute of Genetics and Developmental Biology, Chinese Academy of Sciences, Beijing, China; ^2^University of Chinese Academy of Sciences, Beijing, China

**Keywords:** ethylene, agronomic traits, rice, crops, stress

## Abstract

Gaseous hormone ethylene has diverse effects in various plant processes. These processes include seed germination, plant growth, senescence, fruit ripening, biotic and abiotic stresses responses, and many other aspects. The biosynthesis and signaling of ethylene have been extensively studied in model *Arabidopsis* in the past two decades. However, knowledge about the ethylene signaling mechanism in crops and roles of ethylene in regulation of crop agronomic traits are still limited. Our recent findings demonstrate that rice possesses both conserved and diverged mechanism for ethylene signaling compared with *Arabidopsis*. Here, we mainly focused on the recent advances in ethylene regulation of important agronomic traits. Of special emphasis is its impact on rice growth, flowering, grain filling, and grain size control. Similarly, the influence of ethylene on other relevant crops will be compared. Additionally, interactions of ethylene with other hormones will also be discussed in terms of crop growth and development. Increasing insights into the roles and mechanisms of ethylene in regulating agronomic traits will contribute to improvement of crop production through precise manipulation of ethylene actions in crops.

## Introduction

Ethylene is a simple gaseous phytohormone present in plants and regulates plant growth and developmental processes ranging from germination to senescence (Bleecker and Kende, 2000). Dark-grown *Arabidopsis* seedlings treated with saturated ethylene exhibit triple response that consists of inhibiton of hypocotyls and roots and exaggeration of the curvature apical hooks (Guzmán and Ecker, 1990). Significant progress has been made in the ethylene signal pathway after the discovery of ethylene-insensitive and constitutive ethylene response mutants from the dicotyledonous model *Arabidopsis*. In *Arabidopsis*, ethylene is perceived by its five receptors (Hua and Meyerowitz, 1998). Then receptors and another negative regulator, CONSTITUTIVE TRIPLE-RESPONSE1 (CTR1) are inactivated, and the central signal transducer ETHYLENE-INSENSITIVE 2 (EIN2) C-terminal end (CEND) (Lin et al., 2009a) is dephosphorylated and cleaved. The cleaved CEND is translocated into the nucleus (Qiao et al., 2012; Wen et al., 2012) and the P-body. EIN2 CEND mediates translation repression of ETHYLENE-INSENSITIVE (EIN3) Binding F-Box1/2 (EBF1/2) at P-body and consequently activates ethylene response (Li et al., 2015). In the nucleus, the master transcription factors EIN3 and ETHYLENE INSENSITIVE-LIKE 1 (EIL1) lead to the ethylene-induced transcription activation and ethylene response (Chao et al., 1997). Interestingly, the EIN2 CEND also contributes to the downstream signaling through elevating the acetylation at H3K4 and H3K23 (Zhang et al., 2016a). Although these studies shed better light on how ethylene regulates plant growth and development and adaption to environment, there are still limitations due to the difference between dicotyledonous and monocotyledonous plants.

**FIGURE 1:**
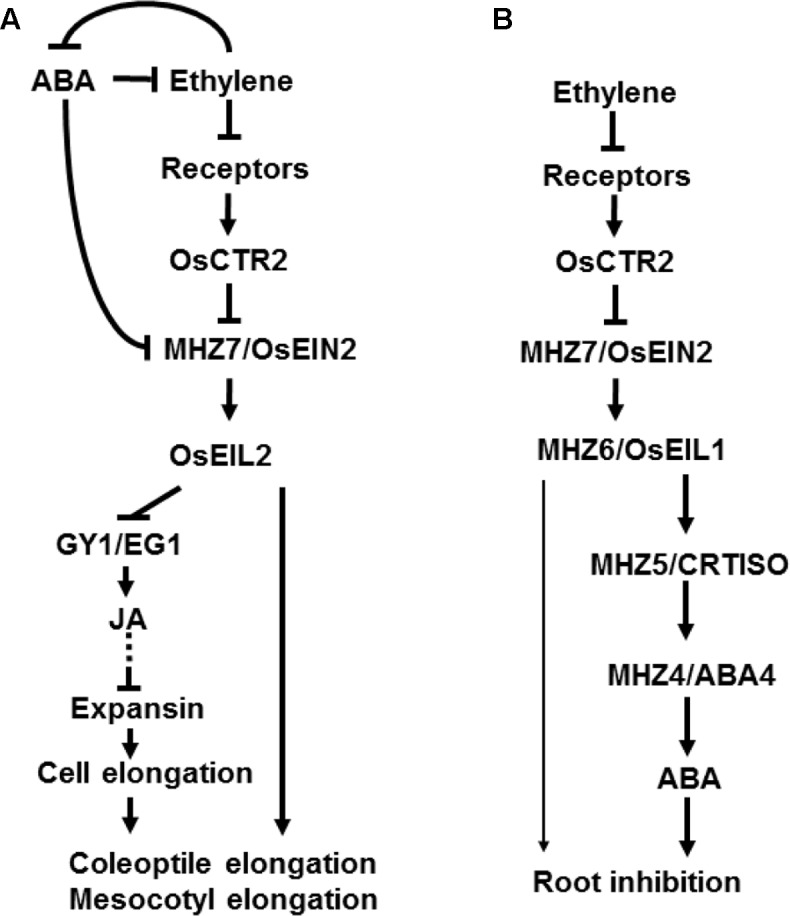
A diagram of ethylene signal transduction pathway in rice. In the dark, rice etiolated seedlings have “double response” with ethylene treatment. Ethylene promotes coleoptile elongation but inhibits root growth in rice. A linear ethylene signaling pathway has been found in rice etiolated seedlings which is similar to that in *Arabidopsis*. Rice has homologs of *Arabidopsis* ethylene signaling pathway, such as ethylene receptors, OsCTRs, MHZ7/OsEIN2, and MHZ6/OsEIL1 and OsEIL2. In contrast to that in *Arabidopsis*, the AtEIN3/EIL1 homologs OsEIL1 and OsEIL2 have divergent functions in rice coleoptile and root growth. **(A)** Ethylene promotes coleoptile/mesocotyl elongation through OsEIL2 which inhibits GY1/EG1-mediated jasmonate (JA) biosynthesis. JA pathway acts downstream of ethylene signaling pathway to inhibit cell elongation. On the other hand, MHZ5/CRTISO and MHZ4/ABA4-mediated ABA pathway acts upstream of ethylene signaling pathway to inhibit transcription of *MHZ7/OsEIN2* and inhibit coleoptile elongation. **(B)** Ethylene regulates root growth through MHZ6/OsEIL1 function. In *Arabidopsis*, AtEIN3 and AtEIL1 have functional redundancy in root inhibition. Moreover, ethylene inhibits root growth partially through MHZ5/CRTISO and MHZ4/ABA4-mediated ABA pathway. Arrows and T-bars indicate direct or indirect activation and inhibition, respectively. Dotted lines indicate several steps involved that are not shown in the diagram.

Semi-aquatic rice has a “double response” (inhibition of root growth but promotion of coleoptile elongation) of dark-grown seedling upon ethylene treatment (Ma et al., 2013). The ethylene growth response kinetics in monocots, including millet, barley and rice, is distinct from that in the dicots such as *Arabidopsis* and tomato (Kim et al., 2012). There are five ethylene receptors in rice genome. OsERS1 and OsERS2 belong to subfamily I with a conserved histidine kinase domain, whereas OsETR2, OsETR3, and OsETR4 belong to subfamily II with a diverged kinase domain (Yang et al., 2015a). But rice and many other monocotyledonous plants do not have ETR1-type ethylene receptor and OsETR2 have Ser/Thr kinase activity (Wuriyanghan et al., 2009). In rice, OsRTH1, OsRTH2, and OsRTH3 are identified and they are homologous to AtRTH1 in *Arabidopsis*. OsRTH1 and AtRTE1 have the highest sequence identity, and ectopic transgenic analysis indicates that only OsRTH1 is able to mimic the function of AtRTE1 (Zhang et al., 2012). AtCTR1 is encoded by a single gene in *Arabidopsis*. Its loss-of-function mutation leads to a constitutive ethylene response (Huang et al., 2003). In rice, three homologs *OsCTR1, OsCTR2* and *OsCTR3* were identified, and OsCTR2 is most closely related to AtCTR1 (Wang et al., 2013; Yang et al., 2015a). Similar to that in *Arabidopsis*, OsEIN2 is a positive regulator in ethylene signal pathway of rice (Ma et al., 2013). EILs are the master transcription factors in ethylene signaling pathway, with six members found in rice (Yang et al., 2015a). However, only OsEIL1 and OsEIL2 regulate root and coleoptile ethylene response respectively, with the left members having no role in ethylene response (Yang et al., 2015b). These results indicate that OsEIL1 and OsEIL2 have divergent function in growth and development in rice. Moreover, both OsEIL1 and OsEIL2 negatively affect rice salt tolerance by promoting transcription of *HIGH-AFFINITY K^+^ TRANSPORTER 2;1* (*HKT2;1*) and Na^+^ absorption in roots (Yang et al., 2015b). Carotenoid isomerase (CRTISO) catalyzes the conversion of prolycopene to all-*trans-*lycopene (Fang et al., 2008). ABA4 drives the conversion of zeaxanthin to neoxanthin but no enzyme activity is detected in *Arabidopsis* (North et al., 2007). Its homologous gene *MHZ4/ABA4* mutation reduced abscisic acid (ABA) production in rice (Ma et al., 2014). Both MHZ5/CRTISO and MHZ4/ABA4 are involved in ABA biosynthesis. The study of the two ethylene response mutants *mhz4/aba4* and *mhz5/crtiso* indicates that ethylene inhibits root growth through ABA pathway in rice (Ma et al., 2014; Yin et al., 2015). In contrast, in *Arabidopsis*, ethylene inhibition of root elongation is ABA-independent (Beaudoin et al., 2000). Besides, MHZ5/CRTISO and MHZ4/ABA4-mediated ABA pathway inhibits rice coleoptile elongation likely through downregulating ethylene signaling pathway (Ma et al., 2014; Yin et al., 2015). These studies indicate that the ethylene signal transduction has conserved and divergent aspects between *Arabidopsis* and rice (**Figure 1**). Furthermore, rice ethylene response is also different from other monocots such as maize, wheat, sorghum, and *Brachypodium distachyon* (Yang et al., 2015a). Therefore, identification of rice ethylene signal transduction components and analysis of the interactions between ethylene and other phytohormones will shed better light on how ethylene is specifically involved in rice growth and adaptation.

**FIGURE 2:**
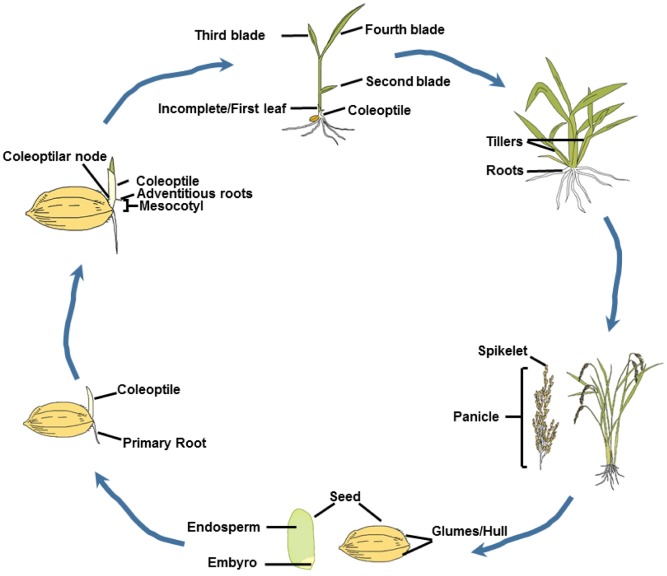
Rice life cycle. The life cycle of rice begins with a seed. The hull is a protective coat of the seed. Endosperm is the starchy part. Both the starch and protein are the determining factor of seed dry weight and seed size. The embryo consists of the precursor tissues of leaves, coleoptile/mesocotyl and roots, and forms the rice seedling. The coleoptiles and mesocotyls play a pivotal role for rice seedling emerging from soil. The roots anchor the plant and absorb nutrients from the soil. The tillering stage is also referred to the late vegetable stage. At this stage, a number of reproductive stems will grow and ultimately produce a set of flower heads, which are referred to spikes. After pollination, the flowers will form seeds. In the rice panicle, superior-spikelets that anthered earlier produce better filling grains than the inferior-spikelets.

Rice, as a major staple crop, feeds more than half of the world’s population (Yu et al., 2016). To satisfy the increasing global demand of the growing population, a 50% increase in rice production will be required (Alexandratos and Bruinsma, 2012). Rice shares close synteny and collinearity with other important cereal crops (Gale and Devos, 1998). Rice has the smallest genome of the major cereals and rich genetic diversity (Sasaki et al., 2005). In addition, the sequence of rice entire genome (Sasaki et al., 2005; Du et al., 2017) provides basis for identification of homologous genes for other crops. Rice is an annual grass, and can finish a life cycle (**Figure 2**) in 5 months or less. Ethylene regulates several stages of life cycle of rice.

Previous research indicates that three phytohormones including gibberellins (GA), cytokinins, and brassinosteroids regulate several agronomically important traits of rice, such as plant height (Sasaki et al., 2002), grain number (Ashikari et al., 2005) and leaf erectness. Ethylene plays a subtle role in plant growth, particularly in adaption to stressful environmental conditions (Peng et al., 2014; Tao et al., 2015). Abiotic and biotic stresses affect growth of crops at various stages and ultimately result in loss of yield. Reproductive processes including flowering, grain filling, and maturation are highly affected by abiotic stresses. Ethylene-insensitive mutants offer a great opportunity for understanding ethylene signal transduction in rice (Ma et al., 2013; Yang et al., 2015b; Yin et al., 2015). Then, how does the ethylene signaling affect agronomic traits? Ethylene impacts on fruit ripening have been studied in a series of plant species, including tomato and apple, etc. (Alexander and Grierson, 2002; Lee et al., 2010; Li et al., 2017). Jasmonate (JA) promotes ethylene biosynthesis to promote apple fruit ripening (Li et al., 2017). This review will highlight the impact of ethylene on crop (especially for rice) agronomic traits, emphasizing that the topics should be further investigated.

## Ethylene and Seedling Growth

Ethylene has a biphasic role, stimulating and inhibiting growth depending on the species, organs/tissue, developmental stages, and environmental conditions (Hattori et al., 2009; Zhong et al., 2012; Yu et al., 2013). In *Arabidopsis*, ethylene inhibits hypocotyl elongation through activating the transcription factors WAVED-DAMPENED 5 (WDL5) (Sun et al., 2015) and ETHYLENE RESPONSE FACTOR 1 (ERF1) (Zhong et al., 2012; Shi et al., 2016a,b) in the dark or low light intensities. Transcription factor HYPOCOTYL 5 (HY5) also participates in this process which is degraded by the E3 ligase CONSTITUTIVE PHOTOMORPHOGENIC 1 (COP1) (Osterlund et al., 2000). In the light, AtEIN3 acts upstream of COP1 to influence COP1 localization and HY5 stabilization, and balances the equilibrium between ethylene and light signaling and ultimately promotes hypocotyl growth (Yu et al., 2013; Shi et al., 2016a,b). These analyses indicate that ethylene signaling pathway is indispensable for plant emergence from the soil.

In rice, coleoptile and mesocotyl are essential protection structures for seedling emergence from the soil. Ethylene promotes coleoptile elongation not only in the dark but also in the light (Ma et al., 2013; Wang et al., 2013; Yin et al., 2015). This fact is different from that in *Arabidopsis* where ethylene plays an opposite role in hypocotyl elongation in dark and in light. In addition, ethylene promotes the curvature of rice coleoptile in the light (Wang et al., 2013). Obviously, identification and characterization of the component of ethylene signaling pathway in rice will benefit us in unraveling its mechanisms in rice. Progress has been made in the function of ethylene on rice growth and development and agronomic traits using the forward and reverse genetic approaches.

The rice ethylene receptor loss-of-function mutants *Osers2, Osetr2*, and *Osetr3* in Zhonghua background all exhibited mild enhanced ethylene response in coleoptile elongation (Wuriyanghan et al., 2009). However, loss-of-function *Osers1, Osers2*, and *Osetr2* mutants in the strain Dongjin (DJ) background showed no apparent phenotype in coleoptile but an enhanced ethylene response in root growth inhibition (Ma et al., 2014; Yin et al., 2015). These results suggest that the rice ethylene receptors may have functional redundancy and background/subspecies-specific features. In *Arabidopsis*, the ethylene insensitivity phenotype of REVERSION-TO-ETHYLENE SENSITIVITY 1 (RTE1) overexpression lines depends on ETR1 (Zhou et al., 2007). The *OsRTH1* overexpression lines exhibited ethylene insensitivity in coleoptile elongation in dark-grown seedlings and coleoptile curvature in light-grown seedlings (Zhang et al., 2012). As the key negative component, CTR’s loss of function mutant *Osctr2* had a stronger exaggerated coleoptile curvature on ethylene treatment and this phenotype is probably not due to different ethylene production but resulted from enhanced ethylene sensitivity (Wang et al., 2013). Loss-of-function mutant *Osein2/mhz7* exhibited complete coleoptile and root insensitivity to ethylene. The *OsEIN2*-overexpression lines showed constitutive ethylene response without ethylene treatment and enhanced ethylene response in the presence of ethylene (Ma et al., 2013). *OsEIL2*-RNAi transgenic lines exhibited coleoptile insensitivity to ethylene, and loss of function in *Oseil1/mhz6* led to ethylene insensitivity in roots (Yang et al., 2015b). These features indicate that OsEIL1/MHZ6 and OsEIL2 have divergent functions in seedling growth in rice, which is different from those in *Arabidopsis* where EIN3/EIL1 showed no apparent organ specificity in ethylene responses.

Upon ethylene perception, OsEIL2 directly bound to the promoter of GAOYAO/EXTRA GLUME (GY1/EG1) (Zhang et al., 2016b) and inhibited GY1/EG1-mediated jasmonic acid biosynthesis and ultimately promoted mesocotyl and coleoptile elongation in etiolated rice seedlings (Xiong et al., 2017). In addition, the promotion of ethylene responses in the coleoptile is correlated with a decrease in the levels of *OsEIL2* and an increase in rice F-box protein *OsEBF2* transcripts upon treatment with 10 ppm ethylene for 3 h, whereas no significant change was observed in the transcript levels of *OsEIL1* (Wang X. et al., 2009; Kim et al., 2012). Whether OsEIL2 exerts its effects on mesocotyl/coleoptile growth through regulation at levels of transcriptional repression activity, its own gene transcription and/or protein degradation need further investigation in rice. Besides, the MHZ4/ABA4 and MHZ5/CRTISO-mediated ABA pathway likely inhibits coleoptile elongation through decreasing the transcription of *OsEIN2* and acts upstream of ethylene signaling pathway (Ma et al., 2014; Yin et al., 2015).

Taken together, the crosstalk between ethylene and light signaling pathway is extensive and complex in plant emergence from soil (Shi et al., 2016a,b; Xiong et al., 2017; Yu and Huang, 2017). The growth of rice coleoptile plays a vital role in early-stage rice seedling growth and development, and ethylene has a major role in this process. What is the molecular mechanism of ethylene in promoting rice coleoptile elongation through OsEIL2? Are there any specific downstream factors, e.g., OsERFs, involved in this process? How does ethylene collaborate with light in regulating these processes? Are the mechanisms different from that in *Arabidopsis*? Revealing the molecular mechanism of ethylene control on the elongation of rice coleoptile/mesocotyl will provide new genetic resources and theoretical basis for breeding new cultivars suitable for growth in dry land.

In addition to regulating etiolated seedlings, ethylene influences rice seedling growth in light. *OsRTH1* overexpression had an inhibitory effect on ethylene-induced leaf elongation and adventitious root growth in rice (Zhang et al., 2012). *Osctr2* produced more adventitious root regardless of ethylene than control cultivar DJ (Wang et al., 2013). When grown in water, compared to the curved roots in control seedlings, the root of *Osein2/mhz7* mutant was longer and straight, while the shoots of *Osein2/mhz7* were significantly shorter than control cultivar (Ma et al., 2013). This short shoot phenotype is consistent with that observed in *OsEIN2*-RNAi plants by Jun et al. (2004). The phenotype of *Oseil1/mhz6* seedling was similar to that of *Osein2/mhz7* mutants above. However, it should be noted that, at mature stage, the plant height of *Oseil1/mhz6* mutant, *OsEIL1*-overexpressing plants and *OsEIL2*-RNAi lines showed no significant difference compared to control plants, while the *OsEIL2*-overexpressing transgenic plants were apparently shorter than control plants (Yang et al., 2015b). The *mhz4/aba4* mutant plants were taller than WT and *MHZ4*-overexpression transgenic lines were shorter than WT. Double mutant analysis revealed that MHZ4/ABA4 negatively regulates plant height in an OsEIN2-dependent manner (Ma et al., 2014). In contrast, the plant height of *mhz5/crtiso* is shorter than WT due to the shorter length of all internodes (Yin et al., 2015). Double mutant analysis revealed that MHZ5/CRTISO regulate plant height in an OsEIN2-independent manner.

## Ethylene and Flowering

The control of plant flowering time is essential to produce sufficient seed for propagation and can be influenced by environmental factors (e.g., day length, temperature, etc.) and endogenous developmental cues of plants. The major genes controlling flowering time are conserved and divergent between *Arabidopsis* and rice (Higgins et al., 2010). The flowering time regulatory networks of the two model plants were compared in the review provided by Shrestha et al. (2014). The two evolutionarily distant plant species had different photoperiod requirement. The B-type response regulator EARLY HEADING DATE 1 (*EHD1*) in rice has no ortholog genes in *Arabidopsis* and it acts independent of HEADING DATE1 (Hd1). This is a unique flowering pathway in rice (Doi et al., 2004).

Ethylene regulates plant flowering (Abeles et al., 1992; Iqbal et al., 2017). In *Arabidopsis*, the ethylene-overproducer mutant *eto1* exhibited early flowering, whereas the dominant gain-of-function *etr1* mutants, and loss-of-function *ein2-1* and *ein3-1* mutants showed delayed flowering (Ogawara et al., 2003). However, the loss-of-function mutation of the key negative regulator Ser/Thr kinase gene *AtCTR1* led to delayed flowering (Achard et al., 2007). The discrepancy of the role of ethylene signaling components in flowering requires further investigation. In rice, the *OsETR2*-RNAi lines and loss-of- function *Osetr2* mutant exhibited early flowering and heading time, and the latter showed enhanced ethylene sensitivity compared to that of control. Overexpression of *OsETR2* transgenic lines exhibited reduced ethylene sensitivity and showed late flowering time compared with that of WT. The delayed flowering is associated with the higher transcript level of *OsGI* (*OsGIGANTEA*) and *RCN1* (*TERMINAL FLOWER1/CENTRORADIALIS-like*). Recessive loss-of-function *Osetr3* mutant also showed early flowering (Wuriyanghan et al., 2009). At mature stage, the *OsETR2*-overexpression lines matured later than controls and RNAi-lines (Wuriyanghan et al., 2009). Both loss-of-function *Osctr2* mutants and the *35S:OsCTR2^*1-513*^* lines exhibited late flowering compared with the control plants (Dongjin and ZH11) (Wang et al., 2013). The panicles of *Osein2/mhz7* mutant seemed to have more green grains and matured later than control plants (Ma et al., 2013). These studies indicate that ethylene signaling likely leads to early flowering in *Arabidopsis* and in rice. Alterations of ABA-biosynthesis pathway in *mhz4/aba4*, *MHZ4*-overexpressing plants, and *mhz5/crtiso* resulted in delayed heading time in comparison with control plants (Ma et al., 2014; Yin et al., 2015).

Ethylene affects plant flowering time by interaction with other classical phytohormones. It has been reported that the inhibitory effect of auxin in *Pharbitis nil* flowering results from the stimulation of ethylene production (Kesy et al., 2008). It is likely that ethylene inhibits flowering probably depending on the ABA level which is influenced by ethylene in *Pharbitis nil* (Wilmowicz et al., 2008). In *Arabidopsis*, gibberellin plays an important role in initiating flowering through promoting specific transcription factors under short days and long days, respectively (Wilson et al., 1992; Porri et al., 2012). DELLA-domain proteins are transcription factors and function to repress gibberellin (GA) responses in plants (Fleet and Sun, 2005). In *Arabidopsis*, ethylene promotes EIN3 accumulation and results in delayed flowering in a DELLA-dependent manner under short day conditions (Achard et al., 2007). These findings suggest that mechanism of ethylene regulation of plant flowering time is complex and needs further investigation.

## Ethylene and Grain Filling

Seed maturation process usually involves grain filling and fruit ripening. Previous studies mainly focus on the mechanism of ethylene on climacteric plant fruit ripening (Barry and Giovannoni, 2007). Ethylene has an auto-inhibitory effect during vegetative growth and an auto-stimulatory effect during fruit ripening (Lelièvre et al., 1998). However, ethylene effect on grain filling of crops is largely obscure and needs elucidation. Rice grain mainly consists of starch, protein, and other metabolites. The two major reserves of starch and protein largely determine the quality and yield of rice (Duan and Sun, 2005). Increasing the number of spikelets per panicle would likely expand yield sink capacity but might result in poor grain filling and low yield. Effect of ethylene on filling of compact-panicle and lax-panicle in rice has been studied. The differential grain filling of the superior and inferior spikelets probably resulted from the higher content of ethylene in compact-panicle rice cultivar (Yang et al., 2006b; Panda et al., 2015, 2016). The basal spikelets produce more ethylene at anthesis, and the expression of ethylene receptors and ethylene signaling transducers retained longer at post-anthesis compared to apical spikelets (Sekhar et al., 2015). Higher ethylene concentration negatively correlated with cell division rate, grain filling and starch concentration, but positively correlated with soluble sugar concentration of the growing endosperm (Panda et al., 2009; Kuanar et al., 2010; Sekhar et al., 2015). Ethylene affects the activity of the enzymes for starch synthesis (Naik and Mohapatra, 2000). The downregulation of essential proteins for rice cell progression and division, such as importin-α, elongation factor 1-β and cell division control protein 48 (CDC48), might limit the sink capacity and lead to poor grain filling in the inferior spikelets compared to that in the superior spikelets (Das et al., 2016). Ethylene negatively regulated grain filling by decreasing the activities of sucrose synthase (SuSase) and starch synthase (StSase) (Zhao et al., 2007). These analyses suggest a negative impact of ethylene on grain filling. Inhibitors including 1-MCP, AVG (aminoethoxyvinylglycine), and STS of ethylene biosynthesis or signaling have been useful in investigating the function of ethylene in rice grain filling. 1-MCP and AVG treatment increased cell number and size, and starch synthesizing enzyme gene expression, and ultimately improved grain filling (Mohapatra and Mohapatra, 2006; Panda et al., 2016). Furthermore, 1-MCP treatment enhanced expression of cell cycle regulators such as *cyclin dependent kinase* (*CDK*), *cyclin* (*CYC*) and *cyclin dependent kinase inhibitor* (*CKI*) and ultimately increased grain filling (Panda et al., 2016).

Using rice lines altered in ethylene signaling, regulation of grain filling by ethylene can be characterized. Ethylene receptor OsETR2 delays the flowering time and influences starch accumulation in the grains through decreasing transcription level of a monosaccharide transporter gene, ultimately leading to a lower seed-setting rate. In addition, both loss-of-function mutants *Osetr2* and *Osetr3* reduced starch accumulation in stems. The seeds of *OsETR2*-RNAi lines ripe earlier than that of seeds from control and overexpression lines (Wuriyanghan et al., 2009). The higher expression level of *OsETR4* at 6 days after anthesis during spikelet development probably correlated with grain-filling (Sekhar et al., 2015). The weight of grains of *Osctr2* is slightly lower than that of DJ plants (Wang et al., 2013). Compared to WT, the total grain weight per plant decreased in *Osein2/mhz7* mutant and *OsEIN2*-overexpression lines (Ma et al., 2013). OsEILs, acting downstream of OsEIN2, induce ethylene responsive factors (ERF) and/or other transcription activation/inhibition events (Hattori et al., 2009; Ma et al., 2013; Yang et al., 2015b). Sekhar et al. (2015) found that *OsEIL1* is negatively associated with grain filling in the inferior spikelets of lax-panicles. Our recent research found that ethylene induced the expression of *MHZ5/CRTISO* and *MHZ4/ABA4* (Ma et al., 2014; Yin et al., 2015). Moreover, grain filling is hindered in *mhz4/aba4* mutant (Ma et al., 2014). Carotenoid metabolism is tightly related to tomato fruit ripening. The ethylene-induced carotenoid biosynthesis may also be beneficial for rice grain filling. These results indicate that alteration of ethylene signaling pathway affects rice grain filling.

Ethylene may work in coordination with other hormones to influence rice/grain filling. Numerous data suggest that ethylene negatively regulates rice inferior grain filling through interaction with ABA and other hormones. Under moderate water-stressed condition, higher ratio of ABA to ethylene/ACC concentrations was beneficial for the grain-filling rate in wheat and rice (Yang et al., 2002b, 2004, 2006a). Our results indicate that mutants of *mhz4/aba4* and *mhz5/crtiso* have decreased ABA contents but higher ethylene production (Ma et al., 2014; Yin et al., 2015). The high ratio of ethylene to ABA inhibits the expression of starch synthesis genes and their enzyme activities and leads to poor grain filling (Zhu et al., 2011). Similar results have also been obtained in wheat. When treated with spermidine and spermine, the endogenous ethylene content decreased and led to poor the grain filling and lower grain weight (Liu et al., 2013). Furthermore, under drought conditions, spermidine and spermine significantly increased zeatin, zeatin riboside and ABA content, decreased ethylene content in grains, and improved grain-filling rate in wheat (Liu et al., 2016). Furthermore, the balance of ethylene and ABA plays a pivotal role in grain-filling rate (Yang et al., 2004). Besides, an increase in ABA content but a decrease in GA content, and the altered balance of hormones in rice grains controlled by moderate water deficit, could enhance the weight of the grains (Yang et al., 2001). Cytokinins accelerate cell division during the early phase of rice grain development and consequently influence grain filling (Yang et al., 2000, 2002a). In addition, ethylene inhibited the filling of grains in the basal spikelets while ABA and auxin stimulated this process (Zhang et al., 2009; Kuanar et al., 2010). IAA content is also associated with cell division at the early grain filling stage (Yang et al., 2001).

Taken together, the grain filling is a complicated process and may be regulated by ethylene and other hormones through interactions (Zhao et al., 2007; Liu et al., 2013, 2016). Roles of the hormones from exogenous application studies and from the endogenous determination studies have their own limitations and advantages. Genetic approaches should be more reliable among these, and mutants and transgenic materials should be more valuable in elucidating roles of hormones in grain filling. However, the molecular mechanisms by which ethylene regulates grain filling are largely obscure and need further investigation.

## Ethylene and Grain Size

As one of the factors determining grain weight, grain size is specified by grain length, grain width, length-to-width ratio, and grain thickness. On the other hand, 1000-grain weight (KGW) is the most reliable trait in assessing grain weight (Huang et al., 2013; Zuo and Li, 2014). Previous studies highlight the pivotal roles of cytokinin (Ashikari et al., 2005), brassinosteroid (BR) (Hong et al., 2003; Tanabe et al., 2005; Sakamoto et al., 2006; Che et al., 2015), and auxin (Ishimaru et al., 2013) on rice grain production. Next the genetic and molecular regulation of ethylene on grain size is discussed.

Rice ethylene response mutants are ideal tools to study the effects of ethylene on agronomic traits. The 1000-grain weights of *OsETR2*-RNAi lines were substantially higher than that of control; however, this parameter was reduced or similar in *ETR2*-overexpression plants (Wuriyanghan et al., 2009). As a central component of ethylene signal transduction, OsEIN2/MHZ7 regulates several agronomic traits and plays a pivotal role in rice production. Compared to WT, the total number of panicles was significantly reduced and the KGW of total grains decreased in OsEIN2/MHZ7-overexpression lines. More precisely, OsEIN2/MHZ7 also affects rice grain size. In four allelic mutants of *mhz7*, the length and width of well-filled grains decreased compared to WT grains except the allelic mutant *mhz7-1*. In *MHZ7*-overexpressing plants, grain length was increased compared to that in WT. Ratio of grain length/grain width in *mhz7* mutants was higher or similar with that of WT (Ma et al., 2013). Similarly, loss-of-function *Oseil1/mhz6* mutant exhibits a significant reduction in grain length and width compared to WT grains. *OsEIL1/MHZ6* overexpression increases grain size and 1000-grain weight (Yang et al., 2015b). The grain size and KGW of *OsEIL2*-OX lines were smaller than those of WT plants whereas the two parameters of the *OsEIL2*-RNAi plants exhibited some fluctuation compared with that of WT (Yang et al., 2015b). From these analyses, we propose that ethylene regulates rice agronomic traits under a subtle control. Substantially, ethylene insensitivity may lead to reduced grain size and grain weight, and enhanced ethylene response may be related to larger grains and/or more grain weight, depending on the individual signaling genes. The seed-setting rate and KGW of *mhz4/aba4* and *mhz5/crtiso* were all significantly reduced. Besides, the two mutants showed an increase in grain length and the mhz4 mutant exhibited a decrease in grain thickness (Ma et al., 2014; Yin et al., 2015). The *mhz5-3 Osein2* double mutant showed significant decreases in both seed-setting rate and 1000-grain weight, which is similar to that of *mhz5/crtiso* mutant plants (Yin et al., 2015). These observations suggest that MHZ5/CRTISO regulates seed-setting rate and 1000-grain weight in rice in an OsEIN2-independent manner.

Compared to other cereals, our knowledge about the molecular mechanism that regulates grain size in rice is more than that of wheat, sorghum and maize (Li et al., 2010; Zuo and Li, 2014). ZmIPT2, an isopentenyl transferase involved in cytokinin biosynthesis, is associated with kernel weight and final grain yield (Weng et al., 2013). Several key genes associated with seed size have been cloned and studied in *Arabidopsis*. AtAP2 is involved in the increase of total protein and seed oil contents and ultimately regulating *Arabidopsis* seed size although it negatively affects plant fertility and growth (Jofuku et al., 2005). The mutation of NIMA-related kinase NEK6 leads to increased ethylene production and rounder seeds, while *AtNEK6* overexpression suppressed expression of several ethylene biosynthesis- and signaling-related genes and resulted in smaller seeds and reduced 1000-grain weight. These results indicate that NEK6 negatively regulates *Arabidopsis* seed size (Zhang et al., 2011). Endosperm is the main component of a rice grain, whereas an *Arabidopsis* seed contains mostly the embryo. This difference suggests that grain size control may be different in various plant species.

## Ethylene and Leaf Senescence

Senescence of vegetative organs is essential for reserve remobilization to developing grain. The main function of leaf senescence is nutrient recycling and this confers an adaptive advantage. To some extent, leaf senescence is beneficial for flowering and seed production. Ethylene is a positive regulator of leaf and flower senescence (Bleecker and Kende, 2000). Studies show that ethylene is one of the most important hormones that affects plant leaf senescence (reviewed by Iqbal et al., 2017). In *Arabidopsis*, the two mutant alleles of *EIN2 oresara2* (*ore2*) and (*ore3*) showed delayed senescence, indicating that ethylene affects developmental leaf senescence (Oh et al., 1997). The AtEIN2 positively accelerates leaf senescence by repressing *microRNA164* (*miRNA164*) and regulating the transcription of ORE1/NAC2. The transcription factor ORE1/NAC2 is one of the targets of *miRNA164* (Kim et al., 2009). *AtEIN3* acts downstream of *AtEIN2* to directly repress the expression of *miRNA164* and increase ORE1/NAC2 gene transcription to promote leaf senescence (Li et al., 2013). In addition, *AtEIN2* affects leaf senescence by regulating the senescence-associated NAC transcription factors (TFs), including ANAC019, AtNAP, ANAC047, ANAC055, ORS1, and ORE1/NAC2. *AtEIN3* acts downstream of AtEIN2 and activates ORE1/NAC2 and AtNAP. Furthermore, ORE1/NAC2 and AtNAP affect leaf senescence by activating transcript of common and/or distinct downstream NAC TF factors (Kim et al., 2014). Recent research indicates that EIN3, ORE1/NAC2 and chlorophyll catabolic gene (CCGs) formed a coherent feed-forward loop in the process of ethylene-regulated chlorophyll degradation (Qiu et al., 2015). These results suggest that ethylene regulation of leaf senescence is a highly intricate process and further studies are required to reveal the role of ethylene in leaf senescence.

It is reported that an F-box protein (containing a Kelch repeat motif) OsFBK12 interacted with *Oryza sativa* S-PHASE KINASE-ASSOCIATED PROTEIN-LIKE PROTEIN (OSK1) to form an SCF complex and degrade its substrate, such as *S*-ADENOSYL-L-METHIONINE SYNTHETASE1 (SAMS1). The degradation of OsSAMS1 results in a lower content of ethylene, ultimately suppressing leaf senescence and increasing seed size and grain number (Chen et al., 2013). Overexpression of OsRTH1 prevented ethylene-induced leaf senescence through repressing the expression of ethylene-inducible gene, such as *submergence 1C (Sub1c), alcohol dehydrogenase 2* (*ADH2*), and *glutathione *S*-transferase* (*SC129*) (Zhang et al., 2012). Loss-of-function *Osctr2* mutant leaves exhibited a much stronger senescence phenotype than Dongjin with or without ethylene treatment. 1-MCP treatment could delay the ethylene-induced senescence in Dongjin but not in *Osctr2* (Wang et al., 2013). *Osein2/mhz7* mutant leaf remained green whereas the overexpression lines turned yellow under the treatment of dark-induced and natural senescence. Some senescence-associated genes (SAGs) were up-regulated, including *OsL36, OsL43, OsL85, OsL55, OsNAC1/2* (Ma et al., 2013). Overexpression of the submergence tolerance gene *SUB1A* delayed dark-induced flag-leaf senescence by limiting ethylene production and responsiveness to the other two positive senescence regulatory hormones JA and salicylic acid (Fukao et al., 2012). Ethylene regulates plant leaf senescence through interactions with other hormones. Ethylene interacts with JA to regulate rice leaf senescence by a direct regulatory cascade of OsCOI1b-OsJAZ-OsEIN3-OsORE1 (Lee et al., 2015). In rice, the *AtNAP* homologous gene *OsNAP* promotes leaf senescence and *OsNAP* is induced by ABA but not 1-aminocyclo-propane-1-carboxylic acid (Liang et al., 2014). These studies suggest that the mechanism of NAC TFs involved in leaf senescence may be different in rice and *Arabidopsis* and need to be further explored.

## Ethylene and Architecture Establishment

Leaf angle is an important morphological trait of plant architecture and influences rice cultivation and grain yield (Sinclair and Sheehy, 1999). Several plant hormones, e.g., ethylene, GA, and auxin are involved in leaf angle formation, and most of these phytohormones regulate the leaf angle through interaction with BRs (Cao and Chen, 1995; Wang L. et al., 2009). In addition, Cao and Chen (1995) suggested that ethylene may be involved in BR-induced rice leaf epinasty. However, Takeno and Pharis (1982) suggest that ethylene is not effective in rice lamina inclination in the intact leaves. Further study is required to reveal the role of ethylene in the BR-induced rice leaf epinasty. Rice ethylene response mutant may be useful for studying the relationship between leaf angle and ethylene. At mature stage, the *OsETR2*-overexpression lines had erect panicles compared with controls and RNAi-lines (Wuriyanghan et al., 2009). *Osein2/mhz7* mutants were more erect than WT plants (Ma et al., 2013).

Great progress has been achieved in identifying important genes associated with rice tiller and panicle branches (for review Liang et al., 2014). Effective tillers produce panicles and play an important role in determining rice yield. The number of panicles per plant is one of the major factors determining rice yield. Previous studies showed that cytokinin (Ashikari et al., 2005), auxin (Xia et al., 2012), and SL (Lin et al., 2009b), and BR (Tong et al., 2009) regulate rice tiller and affect yield. The *OsETR2*-overexpression lines had reduced effective panicles and seed setting rates whereas the RNAi lines had no significant difference compared with the controls (Wuriyanghan et al., 2009). Both *Osctr2* and *35S:OsCTR2^*1-513*^* transgenic lines produced more effective tillers than the wild type (Dongjin and ZH11) (Wang et al., 2013). The ABA-deficient mutant *mhz4/aba4* plants had the same effective tiller number compared to WT, whereas the *MHZ4*-overexpressing lines produced more tillers than WT (Ma et al., 2014). In contrast, the other ethylene abnormal mutant *mhz5/crtiso* had excessive tillers, smaller panicles, and fewer primary and secondary branches in panicles compared with WT plants. The tiller number of double mutant *mhz5-3 Osein2* was the same as *mhz5-3*, indicating that MHZ5/CRTISO regulates plant tiller in an OsEIN2-independent manner (Yin et al., 2015). In addition, the strigolactone biosynthesis is also impaired in *mhz5/crtiso*, and the SL mainly regulates plant tiller/branch (Lin et al., 2009b; Yin et al., 2015). These results suggest that ethylene directly or indirectly regulates rice tiller number.

## Ethylene and Submergence

Ethylene is the primary signal for rice and other semi-aquatic plants to adapt to flooding. Concentration changes of abscisic acid, gibberellin, and auxin are also required to gain fast growth under water (Raskin and Kende, 1984; Cox et al., 2004; Saika et al., 2007; Jackson, 2008). Rice has an abundant genetic diversity and evolved two mechanisms to escape flooding. First, increased ethylene upregulates the expression of *snorkel1/2* (*SK1/2*) via OsEIL1b/OsEIL2 binding to the *SK1* and *SK2* promoters and GA content to promote internode elongation in deepwater rice during flooding (Hattori et al., 2009). Second, SUB1A, an ERF transcription factor existed in limited rice accessions, negatively regulates GA response through restriction of Slender Rice-1 (SLR1) and SLR-like 1 (SLRL1) degradation to inhibit plant elongation during flash floods at the seedling stage in water-tolerance rice (Fukao and Bailey-Serres, 2008). SUB1A promotes plant recovery from drought at the vegetative stage through decrease of leaf water loss and lipid peroxidation, and increase of gene transcript level associated with acclimation to dehydration. In addition, overexpression of SUB1A could augment ABA responsiveness to enhance plant tolerance to drought (Fukao et al., 2011). Fukao and Xiong (2013) have reviewed the intricate regulatory mechanisms of rice plants acclimating the submergence and drought stress, and ethylene plays a key role in this process. Furthermore, the SUB1A is beneficial for plant physiological recovery upon desubmergence due to the greater capability for non-photochemical quenching-mediated photoprotection (Alpuerto et al., 2016). These results suggest that plant had orchestrating regulatory mechanisms to enhance plant adaptation.

## Conclusion and Perspectives

**FIGURE 3:**
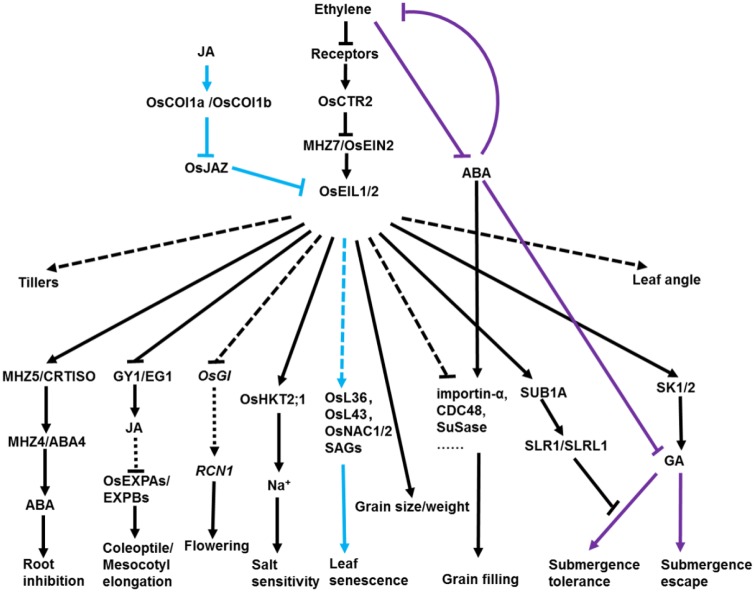
Diverse roles of ethylene in regulating agronomic traits in rice. In rice, ethylene regulates a wide variety of major agronomic traits ranging from emerging from the soil to grain filling and senescence. Ethylene promotes growth of coleoptiles/mesocotyls by partially inhibiting JA biosynthesis. In contrast, JA promotes rice leaf senescence through a cascade of OsCOI1b-OsJAZ-OsEIN3-OsORE1. JA and ethylene synergistically accelerate rice leaf senescence by activating common and differential SAGs. Ethylene positively regulates rice grain size/weight, flowering, tillering and leaf angle but negatively affects rice grain filling and salt tolerance. The lower ratio of ethylene to ABA is beneficial for rice grain filling. Upon submergence, SUB1A negatively regulates GA response by inhibiting the degradation of SLR1/SLRL1 to restrict plant growth in submergence-tolerance species. On the contrary, ethylene promotes internode elongation through increasing transcription of SK1/2 and GA production to escape flooding in deepwater rice. OsGI1, OsGIGANTEA1; RCN1, TERMINAL FLOWER1/CENTRORADIALIS-like; GY1/EG1, GAOYAO/EXTRA GLUME; HKT2;1, HIGH-AFFINITY K^+^ TRANSPORTER2;1; SLR1, Slender Rice-1; SLRL1 SLR-like 1; ABA, abscisic acid; GA, gibberellin; JA, jasmonate; COI1, coronatine insensitive 1; JAZ, jasmonate ZIM-domain protein. Blue lines indicate the leaf senescence regulatory pathway. Purple lines indicate the submergence tolerance regulatory pathway. Arrows and T-bars indicate direct or indirect activation and suppression, respectively. Dotted lines indicate several or unknown steps involved in the pathway.

In this review, we summarized roles of ethylene-related factors and genes in regulation of seedling growth, flowering, grain filling, grain size, leaf senescence, leaf angle, tiller and submergence in rice (summarized in **Figure 3**). At present, several environmental cues, including global warming, heat stress, drought, chilling and salinity, affect crop productivity drastically and threaten global food security. Ethylene signaling is indispensable for plant response and adaptation. It has been proven that EIN3/EIL1 is the integration node of several hormones in plant growth and development and adaption to biotic and abiotic stresses (Kazan, 2015; Wawrzyńska and Sirko, 2016; Quan et al., 2017). Ethylene promotes salt tolerance in *Arabidopsis* (Lei et al., 2011). However, the research of *Oseil1/mhz6* mutants and *OsEIL2*-RNAi lines indicates that ethylene signaling negatively regulates salt tolerance in rice (Yang et al., 2015b; **Figure 3**). The roles of ethylene in plant response to salinity has been reviewed by Tao and the divergence between *Arabidopsis* and rice in the regulation of salinity response by ethylene probably due to the evolutionary divergence under different growing conditions or different plant species (Tao et al., 2015).

In conclusion, higher concentrations of ethylene impair rice grain filling. Similar results were found in wheat. In mature wheat plants, increased ethylene production is associated with decreased 1000-grain weight and hastened maturity (Beltrano et al., 1999). Down-regulating the ethylene biosynthesis pathway can significantly improve the maize grain yield under abiotic stress (Habben et al., 2014). It should be noted that while higher production of ethylene negatively affects several agronomic traits in wheat and rice, overexpression of *OsEIL1/MHZ6* increased grain size and 1000-grain weight (Yang et al., 2015b). *OsEIN2/MHZ7* overexpression increased grain size but not grain weight (Ma et al., 2013), and *OsETR2*-RNAi lines and loss-of-function mutant *Osetr2* produced higher 1000-grain weight than control (Wuriyanghan et al., 2009). This discrepancy suggests that the ethylene biosynthesis and signaling pathways may have different functions in grain size control or the ethylene effect is governed not only by the ethylene production, but also by the tissue sensitivity to ethylene. These discrepancies may also be due to the fact that different plants/stages/methods were used. Although each study has its own contribution toward a special area, genetic analysis should be regarded as an effective approach. While progress has been made in recent years in understanding the molecular basis of ethylene signaling in rice, many fundamental questions remain unanswered. Further investigation is needed to confirm the effects of ethylene on crop agronomic traits, especially grain/yield-related traits, using ethylene biosynthesis and signaling mutant or transgenic plants with an alteration of hormone action. How does ethylene signaling connect to grain filling, storage accumulation, and grain size control? The integration and crosstalk points should be identified. Elucidation of how ethylene influences rice grain development may contribute to the advancement of comparative biological studies in other cereals, and assist in breeding of novel high-yield/quality cultivars.

## Author Contributions

C-CY, BM, S-YC, and J-SZ conceived the topic. C-CY wrote the manuscript. J-SZ revised the final version. HZ drew the picture of rice life cycle.

## Conflict of Interest Statement

The authors declare that the research was conducted in the absence of any commercial or financial relationships that could be construed as a potential conflict of interest.
